# Maternal RSV vaccination in the absence of a nationally funded programme: inequalities in coverage in Norway, 2024 to 2026

**DOI:** 10.2807/1560-7917.ES.2026.31.44.2600882

**Published:** 2026-11-05

**Authors:** Fredrik Skår, Hinta Meijerink, Margrethe Greve-Isdahl, Anja Bråthen Kristoffersen, Jacob Dag Berild, Suzanne Campbell, Håkon Bøås

**Affiliations:** 1 Norwegian Institute of Public Health, Oslo, Norway

**Keywords:** vaccination coverage, respiratory syncytial virus vaccines, pregnancy, socioeconomic factors, Norway, public health surveillance, epidemiology

## Abstract

Respiratory syncytial virus (RSV) is a leading cause of hospitalisation for lower respiratory tract infection in children. Despite not being included in Norway’s national immunisation programme and requiring out-of-pocket payment, maternal RSV vaccination coverage reached 51% by September 2026. Coverage varied markedly by maternal country of birth, with lower uptake among women born outside Norway. Within Oslo, uptake appeared to reflect district-level socioeconomic factors. These inequalities suggest that lack of government funding is an important driver of unequal protection.

Respiratory syncytial virus (RSV) is a leading cause of acute lower respiratory tract infection and hospitalisation among infants and young children worldwide, with the greatest burden concentrated among infants in the first 6 months of life [1]. In Norway, the maternal RSV vaccine has been available since December 2023 and recommended by the Norwegian Institute of Public Health between gestational weeks 24 and 36 since November 2025 [2]. However, maternal RSV vaccination is not publicly funded and requires a prescription and out-of-pocket expenditure (EUR 230 [3], excluding consultation fees) which may contribute to uneven vaccine uptake. Here, we describe coverage of maternal RSV vaccination in Norway, and to examine vaccination coverage by maternal country of birth and place of residence in Norway.

## Maternal RSV vaccination coverage in Norway

Using the national personal identification number, we linked individual-level data from three registries within Stat19 [4] secure server: (i) the Medical Birth Registry of Norway (MBRN) providing date of delivery, gestational age at delivery, residence in Norway at time of delivery; (ii) the Norwegian Immunisation Registry SYSVAK providing the RSV vaccine code and vaccination date, and (iii) the Norwegian Population Register providing area of residence, and maternal country of birth. Coverage was calculated as the number of women with an RSV vaccination recorded in the SYSVAK during pregnancy, divided by the number of women who delivered, as recorded in the MBRN. We estimated the vaccination coverage among women delivering between 1 January 2024 and 30 September 2026. Data were retrieved on 12 October 2026, and all analyses were done using R (version 4.5.0) [5].

Starting in August 2025, we observed a marked increase in in RSV vaccination coverage among women giving birth. The nationwide RSV vaccination coverage increased from 0.3% in January 2024 to 40% in January 2026. Between March and June, the maternal RSV vaccination coverage declined slightly, to 33–37%, before increasing in July and reaching 51% among women who delivered in September 2026 (Figure 1).

**Figure 1:**
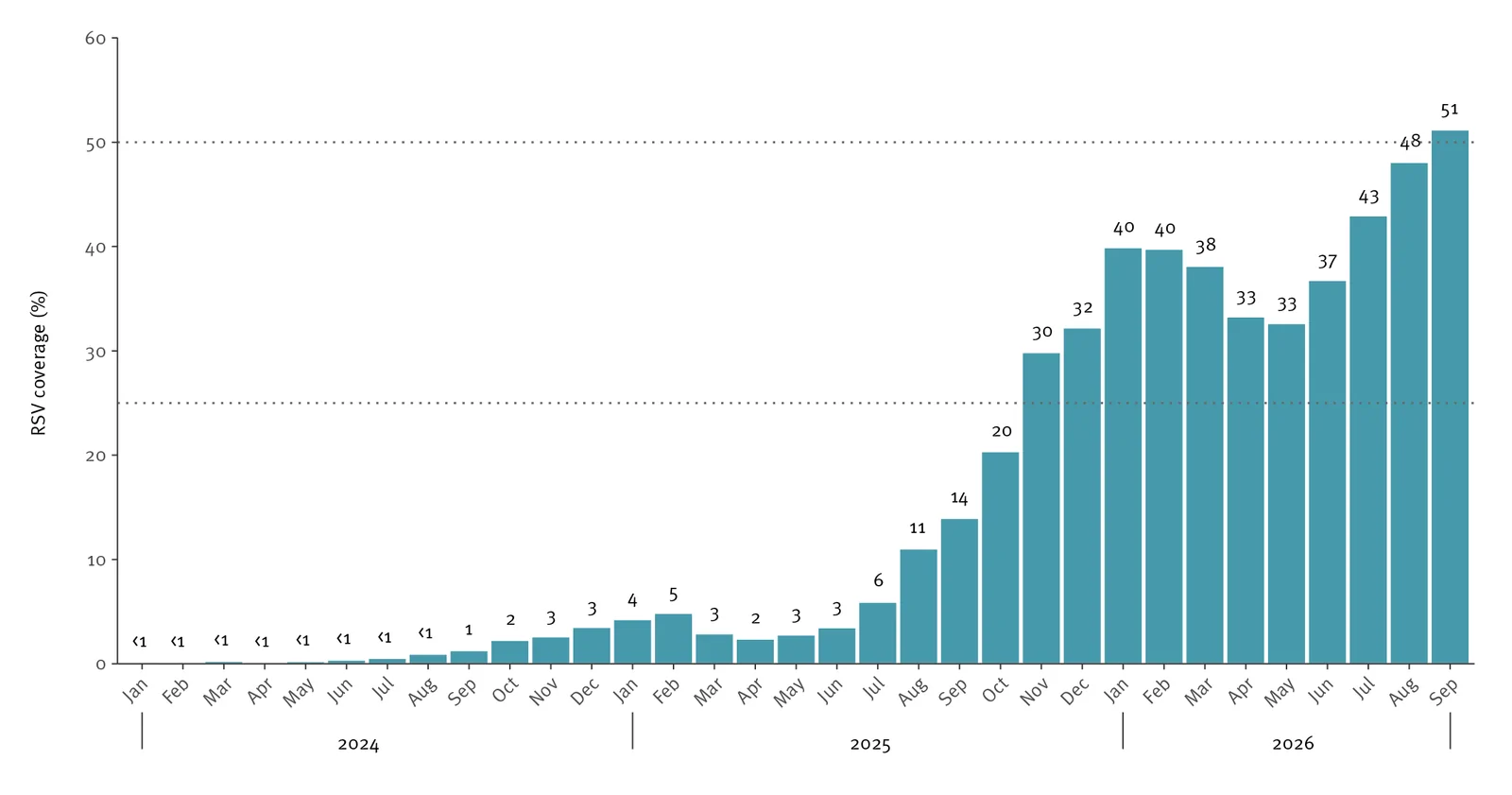
Maternal RSV vaccination coverage in Norway by month of delivery between January 2024 and September 2026 (n = 146,130)

RSV: respiratory syncytial virus.

Coverage was calculated as the number of women with an RSV vaccination recorded in the Norwegian Immunisation Registry (SYSVAK) during pregnancy, divided by the number of women who delivered, as recorded in the Medical Birth Registry of Norway, by month of delivery. Data were linked at the individual level using nationwide registry data. When number of vaccinated women was < 5, the coverage was estimated using 2.5 as the numerator; this was done for January, February and April 2024.

## Maternal RSV vaccination coverage by maternal country of birth

When stratifying RSV vaccination coverage by world region (defined by Statistics Norway [6]), based on the maternal country of birth, we observed that women born in Norway had the highest coverage, reaching 61% among women who delivered in September 2026 (Figure 2). Underlying absolute numbers, coverage and 95% CI are given in Supplementary Table S2. Maternal RSV coverage was lower for women born in outside of Norway, but the monthly coverage followed similar trends as among women born in Norway. The highest monthly coverage between January 2024 and September 2026 by maternal region of birth was 45% for the Americas, compared with 27% for Europe excluding Norway, 21% for Asia and 6% for Africa. (Figure 2).

**Figure 2:**
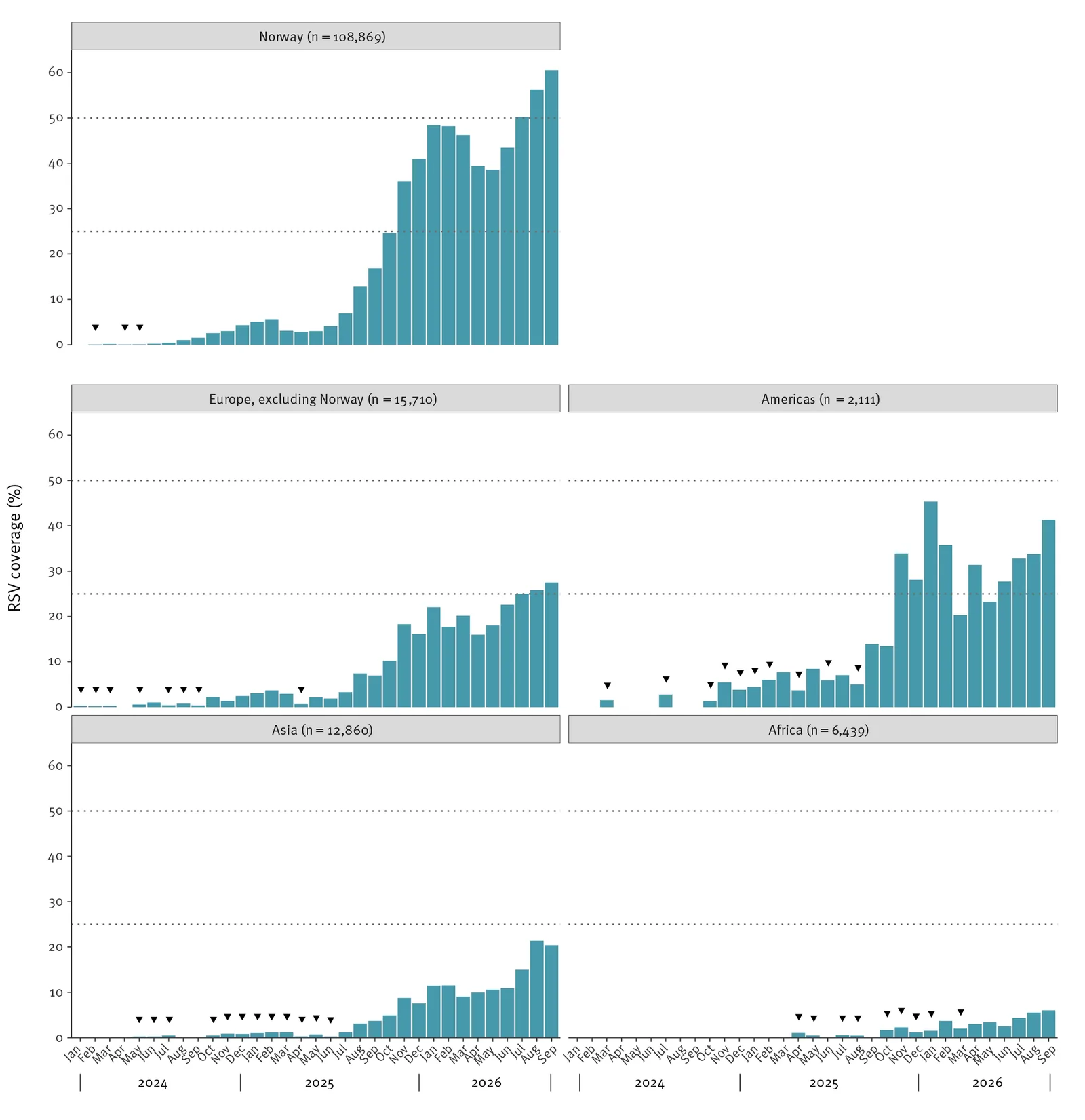
RSV maternal vaccination coverage stratified by world region based on maternal country of birth, among women who delivered in Norway between January 2024 and September 2026 (n = 145,989)

RSV: respiratory syncytial virus.

Coverage was calculated as the number of women with an RSV vaccination recorded in the Norwegian Immunisation Registry (SYSVAK) during pregnancy, divided by the number of women who delivered, as recorded in the Medical Birth Registry of Norway, by month of delivery. Data were linked at the individual level using nationwide registry data. There were too few deliveries from mothers born in Oceania to include in the analysis. Dotted lines show 25% and 50% vaccination coverage. World regions for the analysis are defined by Statistics Norway [6]. When the number of vaccinated was < 5, the coverage was estimated using 2.5 as numerator (months are denoted by a triangle ∇ ).

Restricting to women who delivered between 1 January and 30 September 2026 and including only European countries of origin in which more than 40 women gave birth, the overall cumulative RSV vaccination coverage was 45% (16,282/36,239 women). The coverage was highest among mothers born in Norway (15,412/32,211; 48%), which accounted for 89% of the study population. Combining the European countries by European regions [6], coverage was 38% (309/820) in Nordic countries (Denmark, Finland, Iceland and Sweden; 2.3% of the population) and 33% (239/731) in countries that joined the European Union before 2004, including the United Kingdom (UK) (2% of the population). The coverage was lower among women who were born in countries that joined the EU after 2004 (3.9% of the population) and countries outside the EU/EEA/UK (2.9% of the population) with a coverage of 15% (212/1,422) and 10% (110/1,055) respectively. The division of regions within Europe are based on standards regions used by Statistics Norway [6], and Supplementary Table S4 shows which countries are included in the specified European regions.

The cumulative RSV vaccination coverage among women born in European countries ranged from 6% (Ukraine) to 48% (Norway) (Figure 3). Coverage was highest among those born in the Nordic countries (35–43%), Italy (40%), the UK (38%) and the Netherlands (34%) (Figure 3). An overview of absolute numbers, coverage with 95% CI stratified by maternal European country of birth is provided in Supplementary Table S3.

**Figure 3:**
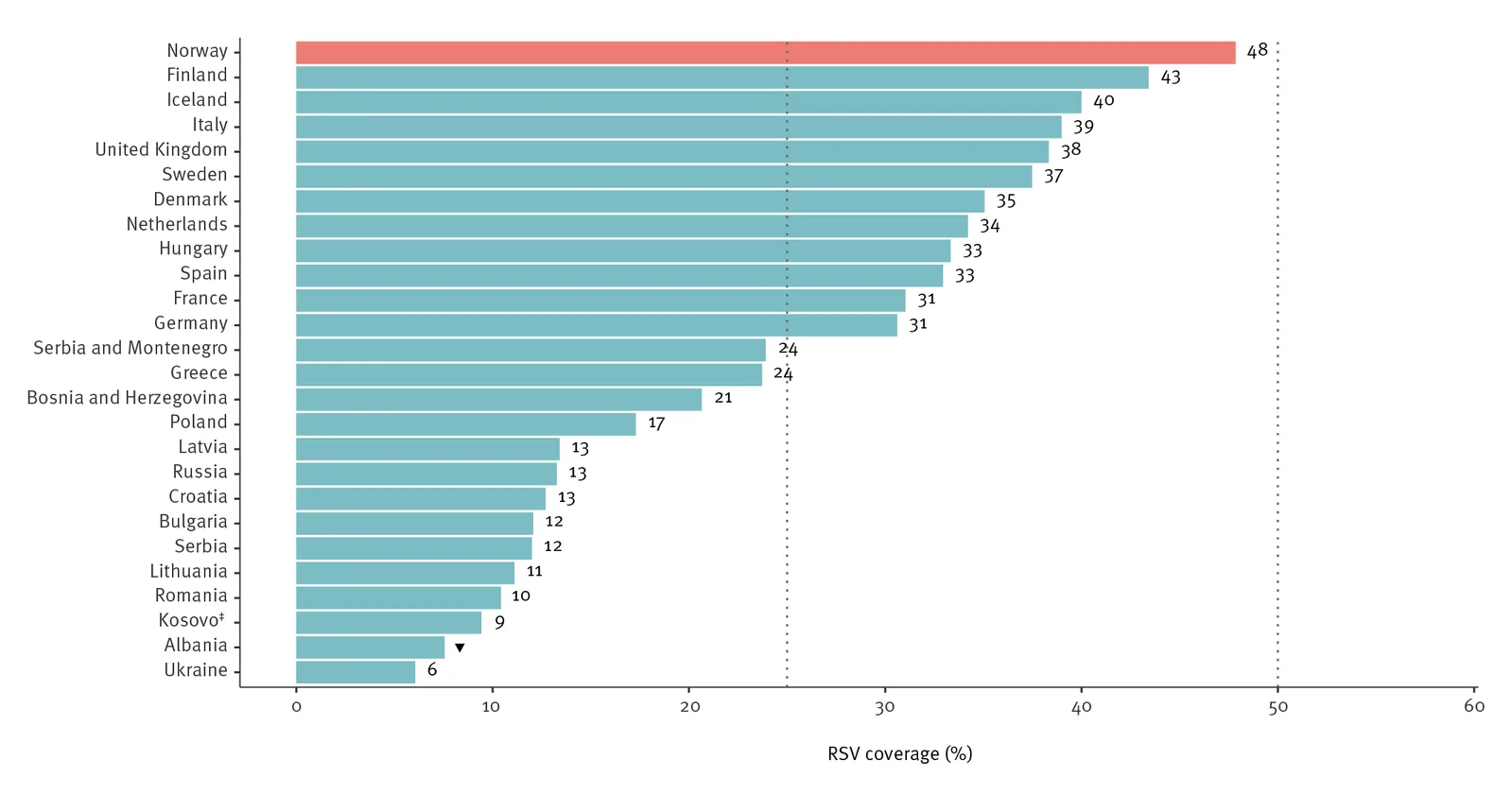
RSV maternal vaccination coverage for women who delivered between January and September 2026, stratified by maternal European country of birth (n = 36,239)

RSV: respiratory syncytial virus.

Coverage was calculated as the number of women with an RSV vaccination recorded in the Norwegian Immunisation Registry (SYSVAK) during pregnancy, divided by the number of women who delivered, as recorded in the Medical Birth Registry of Norway. Data were linked at the individual level using nationwide registry data. Analysis was restricted to countries in Europe with at least 40 deliveries in the specified period. When the number of vaccinated was < 5, the coverage was estimated using 2.5 as numerator (i.e. Albania, denoted by a triangle ∇).

‡ This designation is without prejudice to positions on status and is in line with UNSCR 1244/99 and the IСJ Opinion on the Kosovo declaration of independence.

## Maternal RSV vaccination by geographical area in Norway

Among deliveries from 1 January through 30 September of 2026, there were marked geographical differences in vaccination coverage across Norwegian counties. Coverage ranged from 28% to 50%, with the highest coverage in Oslo county (the county of Norway’s capital city), as shown in Figure 4A.

**Figure 4:**
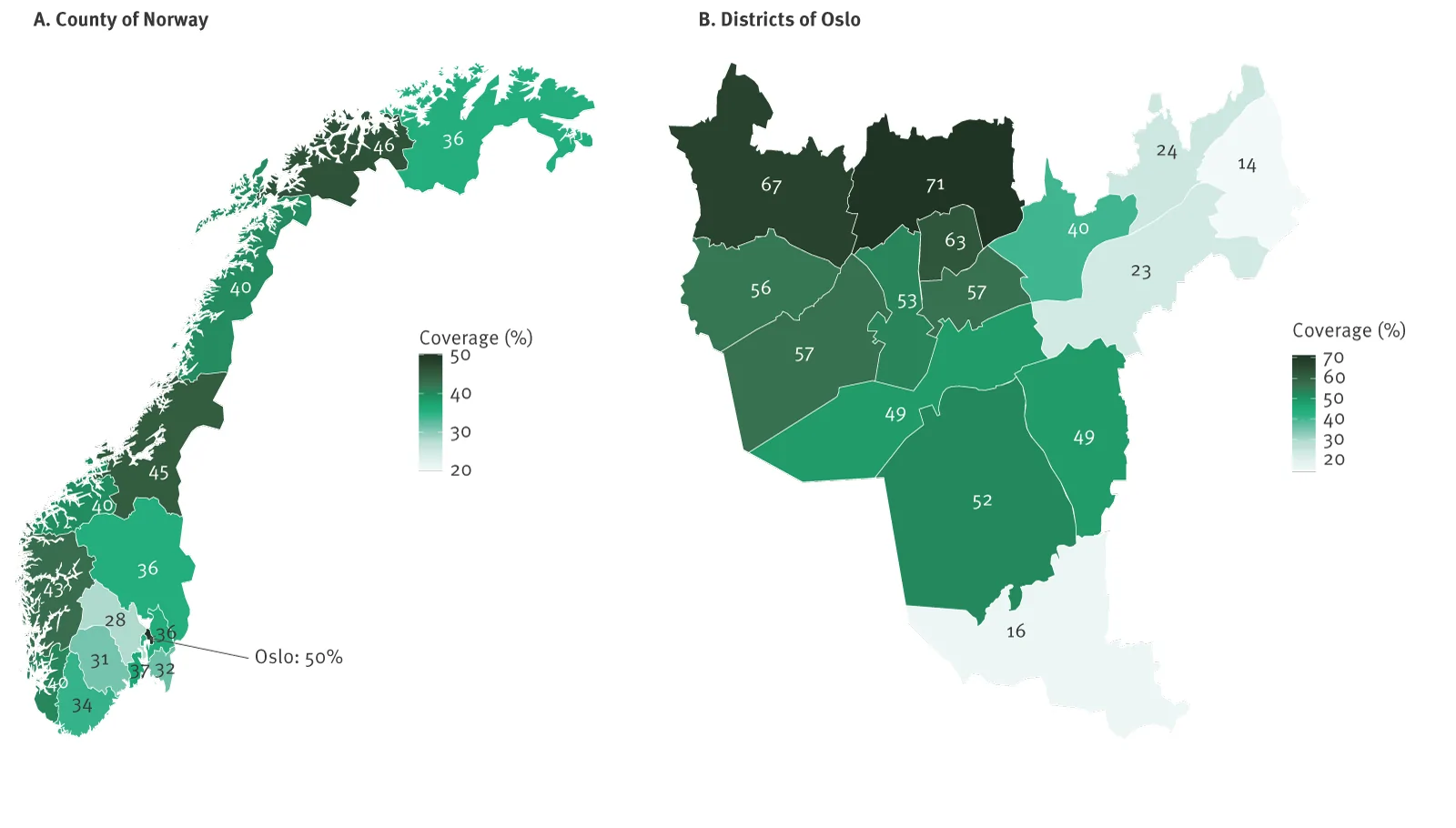
RSV maternal vaccination coverage for women who delivered between 1 January and 30 September 2026, by county of Norway and districts of Oslo (n = 6,928)

RSV: respiratory syncytial virus.

Coverage was calculated as the number of women with an RSV vaccination recorded in the Norwegian Immunisation Registry (SYSVAK) during pregnancy, divided by the number of women who delivered, as recorded in the Medical Birth Registry of Norway between January and September 2026, stratified by (A) county or (B) city districts in Oslo. Data were linked at the individual level using nationwide registry data. Counties of Norway are defined using Nomenclature of territorial units for statistics (NUTS) 3 level. Supplementary Tables S5 and S6 provide the underlying absolute numbers, coverage and 95% CIs.

Within Oslo city, large differences were observed across the city districts, ranging from 14% to 71% (Figure 4B). The distribution of maternal RSV vaccination uptake appeared to follow city district-level socioeconomic factors, including income, education and proportion of migrant residents. Supplementary Figure S1 provides the distribution of household income, higher education and foreign-born individuals by city district level using Statistics Norway data.

## Discussion

Countries that have implemented RSV prevention programmes have demonstrated substantial reductions in RSV-associated hospitalisations [7-9]. Two prevention strategies, passive immunisation of infants with long-acting monoclonal antibodies and maternal vaccination, have been licensed in Norway since 2022 [10] and 2023 [10,11], respectively. This study shows that despite not being included in the national immunisation programme [12], maternal RSV vaccination coverage reached 51% among women who delivered in September 2026 in Norway. Coverage rose considerably from October 2025 to September 2026, coinciding with the Norwegian Institute of Public Health's recommendation of an RSV prevention strategy in November 2025 [2] and substantial media attention on the lack of public funding for the vaccine. However, the coverage varied markedly by maternal country of birth and across counties in Norway. Coverage was lowest among women born in Africa and Asia (6% and 21%, respectively), but substantial variation was also observed among women born in Europe, ranging from 6% to 48%. Oslo county had the highest reported maternal RSV coverage among Norwegian counties. However, we found large differences in uptake by city districts in Oslo, ranging from 14% to 71% among women who delivered between January and September 2026. The coverage followed city district-level socioeconomic factors, with lower coverage in city districts with lower median income, lower education and larger proportion of foreign-born residents.

Vaccination coverage was very low among women born in Africa and Asia. Potential explanations could include socioeconomic disadvantage, shorter duration of residence in Norway, language barriers and limited exposure to information through Norwegian media [13]. As maternal RSV vaccination is not included in the national immunisation programme, information about the recommendation may also be less accessible. In contrast, all Nordic countries were among the top seven maternal countries of birth with highest vaccination coverage. Similarities in language, education, health information and healthcare systems may partly explain the high coverage among Nordic-born women.

Out-of-pocket payment may discourage socioeconomically disadvantaged women. Local variation in how vaccination is discussed and offered may also contribute to differences between municipalities. For vaccines that are not included in a publicly funded immunisation programme, established routines, price and healthcare professionals’ practices are likely to strongly influence whether vaccination is addressed as part of antenatal care [14]. The RSV vaccine requires a prescription by a doctor, and as such women who receive antenatal care exclusively from a midwife may face an additional barrier to access vaccination. Higher coverage is observed in city districts with greater educational attainment, higher income and lower proportion of foreign-born individuals, suggesting that socioeconomic conditions, information access and awareness contribute to vaccine uptake. Oslo county constitutes a single municipality with a relatively small, densely populated geographic area. Antenatal care is organised under the same municipal and national framework across all districts, with the same recommendations and vaccine availability. Differences in access to antenatal care and vaccination services between city districts are therefore expected to be small, so the variation in RSV vaccine coverage is less likely to reflect geographic or organisational barriers and more likely to reflect other factors such as cost.

In October 2026, the Norwegian government announced funding in the proposed 2027 national budget for an RSV prevention programme for infants, using either maternal vaccination or infant monoclonal antibodies depending on the result of a tender [15]. This could potentially alleviate the differences among Norwegian regions and city districts.

A key strength of this study is the use of nationwide, individual-level register data with near-complete coverage of the Norwegian population, linked through unique personal identification numbers, which minimises selection and recall bias and allows robust estimation of vaccination coverage across the entire pregnant population. A limitation is that pregnant women were identified through MBRN, which only captures pregnancies resulting in delivery; women who were still pregnant at the end of the study period are therefore not included.

## Conclusion

We showed substantial variation in RSV vaccine uptake by maternal country of birth, county in Norway and city districts in Oslo pointing to inequalities in access, awareness and likely also socioeconomic factors. The absence of public funding, despite a vaccination recommendation for all pregnant women, is likely an important driver of this inequality. Monitoring whether the socioeconomic differences in uptake observed in this study persist or disappear once the intervention is publicly funded will be important.

## Data Availability

Owing to data privacy regulations in Norway, the raw data cannot be shared. However, data may be obtained from a third party and are available for research upon application through https://helsedata.no/en, after approval from the Norwegian Committee for Medical and Health Research Ethics and within the framework of the Norwegian data protection legislation and any required permission from Authorities.

## ‡ Note

This designation is without prejudice to positions on status and is in line with UNSCR 1244/99 and the IСJ Opinion on the Kosovo declaration of independence.

## Supplementary Data

353000 Supplement

## References

[1] Li Y Wang X Blau DM Caballero MT Feikin DR Gill CJ Global, regional, and national disease burden estimates of acute lower respiratory infections due to respiratory syncytial virus in children younger than 5 years in 2019: a systematic analysis. Lancet. 2022;399(10340):2047-64. 10.1016/S0140-6736(22)00478-0 35598608 PMC7613574

[2] Berild JD, Greve-Isdahl M, Hansen BT, Furuseth E, Bøås H, Fossum E, et al. Anbefaling om immunisering mot RSV for å beskytte spedbarn. Oslo: Folkehelseinstituttet; 2025. Available from: https://www.fhi.no/publ/2025/anbefaling-om-immunisering-mot-rsv-for-a-beskytte-spedbarn

[3] Norwegian Medicines Agency. Abrysvo - 60 micrograms/dose: Norwegian Medicines Agency; 2026. Available from: https://www.legemiddelsok.no/sider/Legemiddelvisning.aspx?pakningId=cfa97b33-829a-4678-9b03-c2213f0b6465&searchquery=abrysvo&f=Han;MtI;Vir;ATC;Var;Mar;Mid;Avr;gen;par;&pane=0r

[4] Norwegian institute of public health. Stat19 – Ett av flere digitaliseringsinitiativ i FHI. [Stat19 – One of Several Digitalisation Initiatives at NIPH.] Oslo: Norwegian institute of public health. [Accessed: 27 Nov 2025]. Norwegian. Available from: https://www.fhi.no/ta/stat19

[5] R Core Team. R: A language and environment for statistical computing. Vienna, Austria: R Foundation for Statistical Computing; 2021. Available from: https://cran.r-project.org/doc/manuals/fullrefman.pdf

[6] Moe D, Molstad C. Standarder for verdensinndelinger og landkoder: Revisjon, dokumentasjon og kartlegging (2022/20). ISBN: 978-82-587-1537-2. [Standards for world regional classifications and country codes: Revision, documentation and mapping (2022/20).] Oslo: Statistisk Sentralbyrå; 2022. Available from: https://www.ssb.no/befolkning/innvandrere/artikler/nye-og-reviderte-standarder-for-verdensinndelinger-og-landkoder/_/attachment/inline/30a23a25-c859-4363-b9ac-86f6c0092bc0:3bcc7282c895573f86d0dbeb87d52ae54dbfdb59/NOT2022-20_rettet-des22.pdf

[7] Jabagi MJ Cohen J Bertrand M Chalumeau M Zureik M. Nirsevimab effectiveness at preventing RSV-related hospitalization in infants. NEJM Evid. 2025;4(3):a2400275. 10.1056/EVIDoa2400275 39998305

[8] Valtuille Z Fafi I Kaguelidou F Levy C Cohen R Mandelbrot L Effectiveness of nirsevimab immunisation after birth versus RSVpreF maternal vaccination in preventing RSV-related hospitalisations in infants: a population-based retrospective cohort study. Lancet Child Adolesc Health. 2026;10(7):481-90. 10.1016/S2352-4642(26)00075-1 42107385

[9] Wadia U Ong MM Cox SN Le H Foo D Wolf Mankiewicz S Maternal RSVpref vaccine effectiveness against respiratory syncytial virus in hospitalized infants. JAMA Pediatr. 2026;e263391. 10.1001/jamapediatrics.2026.3391 42606854 PMC13482632

[10] European medicines agency (EMA). Beyfortus. Amsterdam: EMA; 2023. Available from: https://www.ema.europa.eu/en/medicines/human/EPAR/beyfortus#authorisation-details

[11] European medicines agency (EMA). Abrysvo. Amsterdam: EMA. [Accessed: 10 Oct 2026]. Available from: https://www.ema.europa.eu/en/medicines/human/EPAR/abrysvo

[12] LOVDATA. Forskrift om nasjonalt vaksinasjonsprogram. [Regulations on the national vaccination program]. Oslo: Lovdata; 2009. Norwegian. Available: https://lovdata.no/dokument/SF/forskrift/2009-10-02-1229

[13] Bains S Skråning S Sundby J Vangen S Sørbye IK Lindskog BV. Challenges and barriers to optimal maternity care for recently migrated women - a mixed-method study in Norway. BMC Pregnancy Childbirth. 2021;21(1):686. 10.1186/s12884-021-04131-7 34620114 PMC8495671

[14] Geoghegan S Shuster S Butler KM Feemster KA. Understanding barriers and facilitators to maternal immunization: a systematic narrative synthesis of the published literature. Matern Child Health J. 2022;26(11):2198-209. 10.1007/s10995-022-03508-0 36173503 PMC9521012

[15] Norwegian Ministry of Health and Welfare. Regjeringen vil ha beskyttelse mot RS-virus inn i barnevaksinasjonsprogrammet. [The government wants to include protection against respiratory syncytial virus (RSV) in the childhood immunisation programme.] Oslo: Norwegian Ministry of Health and Welfare. [Accessed: 7 Oct 2026]. Norwegian. Available from: https://www.regjeringen.no/no/aktuelt/regjeringen-vil-ha-beskyttelse-mot-rs-virus-inn-i-barnevaksinasjonsprogrammet/id3174122

