# Supplementary material for: Maternal RSV vaccination in the absence of a nationally funded programme: inequalities in coverage in Norway, 2024 to 2026
Source: Euro Surveill. 2026 Nov 5;31(44):2600882. doi: 10.2807/1560-7917.ES.2026.31.44.2600882 (PMC13743905; doi:10.2807/1560-7917.ES.2026.31.44.2600882)
Supplement: Supplementary file 1 [file 26-00882_SKAR_Supplement.pdf]

Supplementary material for “Maternal RSV vaccination without a nationally funded programme: inequalities in coverage in Norway, 2024 to 2026

Fredrik Skår, Hinta Meijerink, Margrethe Greve-Isdahl, Anja Bråthen Kristoffersen, Jacob Dag Berild, Suzanne Campbell, Håkon Bøås

This supplementary material is hosted by Eurosurveillance as supporting information alongside the article “Maternal RSV vaccination without a nationally funded programme: inequalities in coverage in Norway, 2024 to 2026” on behalf of the authors who remain responsible for the accuracy and appropriateness of the content. The same standards for ethics, copyright, attributions and permissions as for the article apply.

Eurosurveillance is not responsible for the maintenance of any links or email addresses provided therein.

Content

The tables and figures in the document provide the underlaying numbers as well as supplementary results for the article “Maternal RSV vaccination without a nationally funded programme: inequalities in coverage in Norway, 2024 to 2026”.

The following information is included:

Table S1. Maternal RSV vaccination coverage by month of delivery in Norway, January 2024 to September 2026. 2

Table S2. Maternal RSV coverage by month of delivery and maternal region of birth in Norway, January 2024 to September 2026. 3

Table S3. Cumulative maternal RSV vaccination coverage among women who delivered in Norway between 1 January and 30 September 2026, stratified by maternal country of birth in Europe. 7

Tabel S4. Table of countries included in the European Regions, according to Statistics Norway; https://dataportal.ssb.no/classifications/545/codes 8

Tabel S5. Maternal RSV coverage stratified by county of residence at time of delivery for women who delivered between 1 January and 30 September 2026, Norway 9

Table S6. Maternal RSV coverage stratified by district of residence in Oslo at time of delivery for women who delivered between 1 January and 30 September 2026, Norway 10

Figure S1. Overview of socio-economic distribution among Oslo districts based on data from Statistics Norway. Data extracted on 15 October 2026 from https://www.ssb.no/statbank 11

Table S1. Maternal RSV vaccination coverage by month of delivery in Norway, January 2024 to September 2026.

Period	Total	RSV Vaccinated*			
	N	N	%	95%CI	
Jan 2024	3985	<5	<0.13		
Feb 2024	4062	<5	<0.12		
Mar 2024	4316	7	0.16	0.08	0.33
Apr 2024	4300	<5	<0.12		
May 2024	4554	7	0.15	0.07	0.32
Jun 2024	4559	13	0.29	0.17	0.49
Jul 2024	4699	21	0.45	0.29	0.68
Aug 2024	4692	40	0.85	0.63	1.16
Sep 2024	4366	52	1.19	0.91	1.56
Oct 2024	4410	96	2.18	1.79	2.65
Nov 2024	3786	95	2.51	2.06	3.06
Dec 2024	3217	110	3.42	2.84	4.10
Jan 2025	4230	177	4.18	3.62	4.83
Feb 2025	3930	187	4.76	4.14	5.47
Mar 2025	4265	120	2.81	2.36	3.35
Apr 2025	4415	101	2.29	1.89	2.77
May 2025	4728	127	2.69	2.26	3.19
Jun 2025	4639	157	3.38	2.90	3.94
Jul 2025	4966	289	5.82	5.20	6.51
Aug 2025	5017	549	10.94	10.11	11.84
Sep 2025	4665	647	13.87	12.91	14.89
Oct 2025	4477	907	20.26	19.11	21.46
Nov 2025	3873	1153	29.77	28.35	31.23
Dec 2025	3389	1089	32.13	30.58	33.73
Jan 2026	4230	1685	39.83	38.37	41.32
Feb 2026	4106	1629	39.67	38.19	41.18
Mar 2026	4681	1780	38.03	36.65	39.43
Apr 2026	4668	1550	33.2	31.87	34.57
May 2026	5087	1656	32.55	31.28	33.85
Jun 2026	5007	1837	36.69	35.36	38.03
Jul 2026	5157	2211	42.87	41.53	44.23
Aug 2026	5052	2425	48	46.63	49.38
Sep 2026	4602	2352	51.11	49.66	52.55

** When reported numbers were below 5, coverage was calculated based on 5 hypothetical vaccinees and the coverage is therefore presumed to be lower the estimated coverage.*

Table S2. Maternal RSV coverage by month of delivery and maternal region of birth in Norway, January 2024 to September 2026.

Period	Total	RSV Vaccinated*			
	N	N	%	95% CI	
**Norway**					
Jan 2024	2899	0	0.00	0.00	0.13
Feb 2024	2976	<5	<0.17		
Mar 2024	3164	5	0.16	0.07	0.37
Apr 2024	3256	<5	<0.15		
May 2024	3384	<5	<0.15		
Jun 2024	3373	7	0.21	0.10	0.43
Jul 2024	3501	15	0.43	0.26	0.71
Aug 2024	3483	36	1.03	0.75	1.43
Sep 2024	3174	49	1.54	1.17	2.03
Oct 2024	3224	82	2.54	2.05	3.15
Nov 2024	2763	83	3.00	2.43	3.71
Dec 2024	2222	95	4.28	3.51	5.20
Jan 2025	3066	156	5.09	4.36	5.92
Feb 2025	2947	165	5.60	4.83	6.49
Mar 2025	3164	97	3.07	2.52	3.73
Apr 2025	3339	93	2.79	2.28	3.40
May 2025	3561	107	3.00	2.49	3.62
Jun 2025	3506	143	4.08	3.47	4.79
Jul 2025	3787	262	6.92	6.15	7.77
Aug 2025	3847	493	12.82	11.80	13.91
Sep 2025	3482	587	16.86	15.65	18.14
Oct 2025	3345	824	24.63	23.20	26.12
Nov 2025	2815	1014	36.02	34.27	37.81
Dec 2025	2380	975	40.97	39.01	42.96
Jan 2026	3105	1503	48.41	46.65	50.16
Feb 2026	3083	1485	48.17	46.41	49.93
Mar 2026	3517	1626	46.23	44.59	47.88
Apr 2026	3588	1415	39.44	37.85	41.05
May 2026	3886	1499	38.57	37.06	40.12
Jun 2026	3771	1639	43.46	41.89	45.05
Jul 2026	3962	1989	50.20	48.65	51.76
Aug 2026	3852	2168	56.28	54.71	57.84
Sep 2026	3447	2088	60.57	58.93	62.19
**Europe, excluding Norway**					
Jan 2024	442	<5	<1.13		
Feb 2024	478	<5	<1.05		
Mar 2024	451	<5	<1.11		
Apr 2024	443	0	0.00	0.00	0.86
May 2024	513	<5	<0.97		
Jun 2024	492	5	1.02	0.43	2.36
Jul 2024	510	<5	<0.98		
Aug 2024	524	<5	<0.95		
Sep 2024	526	<5	<0.95		
Oct 2024	492	11	2.24	1.25	3.96

Period	Total	RSV Vaccinated*			
	N	N	%	95% CI	
Nov 2024	442	6	1.36	0.62	2.93
Dec 2024	405	10	2.47	1.35	4.48
Jan 2025	488	15	3.07	1.87	5.01
Feb 2025	406	15	3.69	2.25	6.01
Mar 2025	474	14	2.95	1.77	4.90
Apr 2025	461	<5	<1.08		
May 2025	462	10	2.16	1.18	3.94
Jun 2025	477	9	1.89	1.00	3.55
Jul 2025	484	16	3.31	2.04	5.30
Aug 2025	527	39	7.40	5.46	9.96
Sep 2025	502	35	6.97	5.06	9.54
Oct 2025	490	50	10.20	7.83	13.20
Nov 2025	454	83	18.28	15.00	22.10
Dec 2025	421	68	16.15	12.95	19.97
Jan 2026	454	100	22.03	18.46	26.06
Feb 2026	407	72	17.69	14.29	21.69
Mar 2026	486	98	20.16	16.84	23.96
Apr 2026	426	68	15.96	12.79	19.74
May 2026	478	86	17.99	14.81	21.69
Jun 2026	581	131	22.55	19.34	26.12
Jul 2026	504	126	25.00	21.42	28.96
Aug 2026	511	132	25.83	22.23	29.80
Sep 2026	499	137	27.45	23.72	31.53
**America**					
Jan 2024	65	0	0.00	0.00	5.58
Feb 2024	43	0	0.00	0.00	8.20
Mar 2024	66	<5	<7.58		
Apr 2024	62	0	0.00	0.00	5.83
May 2024	46	0	0.00	0.00	7.71
Jun 2024	78	0	0.00	0.00	4.69
Jul 2024	72	<5	<6.94		
Aug 2024	69	0	0.00	0.00	5.27
Sep 2024	68	0	0.00	0.00	5.35
Oct 2024	77	<5	<6.49		
Nov 2024	55	<5	<9.09		
Dec 2024	52	<5	<9.62		
Jan 2025	45	<5	<11.11		
Feb 2025	50	<5	<10		
Mar 2025	65	5	7.69	3.33	16.78
Apr 2025	54	<5	<9.26		
May 2025	71	6	8.45	3.93	17.24
Jun 2025	68	<5	<7.35		
Jul 2025	71	5	7.04	3.05	15.45
Aug 2025	60	<5	<8.33		
Sep 2025	72	10	13.89	7.72	23.71
Oct 2025	67	9	13.43	7.23	23.60
Nov 2025	59	20	33.90	23.14	46.63

Period	Total	RSV Vaccinated*			
	N	N	%	95% CI	
Dec 2025	57	16	28.07	18.08	40.83
Jan 2026	75	34	45.33	34.57	56.55
Feb 2026	56	20	35.71	24.46	48.81
Mar 2026	74	15	20.27	12.69	30.79
Apr 2026	67	21	31.34	21.51	43.20
May 2026	69	16	23.19	14.81	34.40
Jun 2026	65	18	27.69	18.29	39.58
Jul 2026	64	21	32.81	22.57	45.00
Aug 2026	74	25	33.78	24.05	45.12
Sep 2026	75	31	41.33	30.88	52.63
**Asia**					
Jan 2024	378	0	0.00	0.00	1.01
Feb 2024	372	0	0.00	0.00	1.02
Mar 2024	419	0	0.00	0.00	0.91
Apr 2024	364	0	0.00	0.00	1.04
May 2024	397	<5	<1.26		
Jun 2024	404	<5	<1.24		
Jul 2024	408	<5	<1.23		
Aug 2024	409	0	0.00	0.00	0.93
Sep 2024	403	0	0.00	0.00	0.94
Oct 2024	407	<5	<1.23		
Nov 2024	340	<5	<1.47		
Dec 2024	365	<5	<1.37		
Jan 2025	405	<5	<1.23		
Feb 2025	348	<5	<1.44		
Mar 2025	348	<5	<1.44		
Apr 2025	358	<5	<1.40		
May 2025	430	<5	<1.16		
Jun 2025	377	<5	<1.33		
Jul 2025	428	5	1.17	0.50	2.71
Aug 2025	357	11	3.08	1.73	5.43
Sep 2025	407	15	3.69	2.25	5.99
Oct 2025	387	19	4.91	3.17	7.54
Nov 2025	365	32	8.77	6.28	12.11
Dec 2025	357	27	7.56	5.25	10.78
Jan 2026	393	45	11.45	8.67	14.98
Feb 2026	365	42	11.51	8.63	15.19
Mar 2026	397	36	9.07	6.62	12.30
Apr 2026	382	38	9.95	7.33	13.36
May 2026	445	47	10.56	8.04	13.76
Jun 2026	385	42	10.91	8.17	14.42
Jul 2026	441	66	14.97	11.94	18.60
Aug 2026	412	88	21.36	17.68	25.57
Sep 2026	407	83	20.39	16.76	24.58
**Africa**					
Jan 2024	195	0	0.00	0.00	1.93
Feb 2024	188	0	0.00	0.00	2.00

Period	Total	RSV Vaccinated*			
	N	N	%	95% CI	
Mar 2024	214	0	0.00	0.00	1.76
Apr 2024	173	0	0.00	0.00	2.17
May 2024	209	0	0.00	0.00	1.80
Jun 2024	209	0	0.00	0.00	1.80
Jul 2024	204	0	0.00	0.00	1.85
Aug 2024	204	0	0.00	0.00	1.85
Sep 2024	192	0	0.00	0.00	1.96
Oct 2024	207	0	0.00	0.00	1.82
Nov 2024	182	0	0.00	0.00	2.07
Dec 2024	170	0	0.00	0.00	2.21
Jan 2025	219	0	0.00	0.00	1.72
Feb 2025	175	0	0.00	0.00	2.15
Mar 2025	208	0	0.00	0.00	1.81
Apr 2025	196	<5	<2.55		
May 2025	204	<5	<2.45		
Jun 2025	207	0	0.00	0.00	1.82
Jul 2025	188	<5	<2.66		
Aug 2025	219	<5	<2.28		
Sep 2025	199	0	0.00	0.00	1.89
Oct 2025	181	<5	<2.76		
Nov 2025	177	<5	<2.82		
Dec 2025	171	<5	<2.92		
Jan 2026	201	<5	<2.49		
Feb 2026	189	7	3.70	1.81	7.45
Mar 2026	203	<5	<2.46		
Apr 2026	200	6	3.00	1.38	6.39
May 2026	206	7	3.40	1.66	6.85
Jun 2026	199	5	2.51	1.08	5.75
Jul 2026	183	8	4.37	2.23	8.39
Aug 2026	200	11	5.50	3.10	9.58
Sep 2026	167	10	5.99	3.28	10.67

** When reported numbers were below 5, coverage was calculated based on 5 hypothetical vaccinees and the coverage is therefore presumed to be lower.*

Table S3. Cumulative maternal RSV vaccination coverage among women who delivered in Norway between 1 January and 30 September 2026, stratified by maternal country of birth in Europe.

Maternal birth country#	Population		RSV Vaccinated*			
	N	%**	N	%	95%CI	
Total	36239	100	16282	44.93		
Norway	32211	88.88	15412	47.85	47.3	48.39
Finland	76	0.21	33	43.42	32.86	54.61
Iceland	55	0.15	22	40	28.12	53.19
Italy	59	0.16	23	38.98	27.58	51.73
United Kingdom	120	0.33	46	38.33	30.12	47.27
Sweden	515	1.42	193	37.48	33.4	41.73
Denmark	174	0.48	61	35.06	28.36	42.4
Netherlands	76	0.21	26	34.21	24.54	45.4
Hungary	42	0.12	14	33.33	21.01	48.45
Spain	85	0.23	28	32.94	23.88	43.48
France	87	0.24	27	31.03	22.29	41.38
Germany	245	0.68	75	30.61	25.18	36.65
Serbia and Montenegro	46	0.13	11	23.91	13.91	37.94
Greece	59	0.16	14	23.73	14.69	35.97
Bosnia and Herzegovina	92	0.25	19	20.65	13.64	30.02
Poland	688	1.9	119	17.3	14.65	20.3
Latvia	82	0.23	11	13.41	7.66	22.45
Russia	196	0.54	26	13.27	9.22	18.73
Croatia	63	0.17	8	12.7	6.58	23.11
Bulgaria	58	0.16	7	12.07	5.97	22.88
Serbia	100	0.28	12	12	7	19.81
Lithuania	297	0.82	33	11.11	8.02	15.19
Romania	192	0.53	20	10.42	6.84	15.54
Kosovo	106	0.29	10	9.43	5.21	16.5
Albania	53	0.15	4	7.55		
Ukraine	462	1.27	28	6.06	4.23	8.62

*#Limited to countries with >40 deliveries during the included period. * When reported numbers were below 5, coverage was calculated based on 5 hypothetical vaccinees and the coverage is therefore presumed to be lower. **Percentage of total study population included*

Tabel S4. Table of countries included in the European Regions, according to Statistics Norway; https://dataportal.ssb.no/classifications/545/codes

European region	Country
Nordic countries except Norway	Denmark
	Greenland
	Finland
	Faroe Islands (the)
	Iceland
	Sweden
EU/EFTA until 2004 except the Nordics	Andorra
	Austria
	Belgium
	France
	Germany
	Gibraltar
	Greece
	Ireland
	Italy
	Jersey
	Liechtenstein
	Luxembourg
	Monaco
	Netherlands (the)
	Portugal
	San Marino
	Spain
	Switzerland
	United Kingdom of Great Britain and Northern Ireland (the)
New EU countries after 2004	Bulgaria
	Estonia
	Croatia
	Latvia
	Malta
	Poland
	Romania
	Lithuania
	Slovenia
	Hungary
	Slovakia
	Czechia
	Cyprus
Europe except for EU/EFTA/UK	Albania
	Belarus
	Moldova (the Republic of)
	Russian Federation (the)
	Ukraine
	Bosnia and Herzegovina
	North Macedonia
	Serbia
	Montenegro
	Kosova

Tabel S5. Maternal RSV coverage stratified by county of residence at time of delivery for women who delivered between 1 January and 30 September 2026, Norway

County	Population	RSV Vaccinated			
	N	N	%	95% CI	
Oslo	6928	3480	50.23	49.05	51.41
Troms	1165	538	46.18	43.33	49.05
Trøndelag	3679	1653	44.93	43.33	46.54
Vestland	5371	2301	42.84	41.52	44.17
Rogaland	4209	1699	40.37	38.89	41.86
Nordland	1566	625	39.91	37.51	42.36
Møre og Romsdal	1882	747	39.69	37.50	41.92
Vestfold	1680	619	36.85	34.57	39.18
Akershus	5665	2045	36.10	34.86	37.36
Innlandet	2336	835	35.74	33.83	37.71
Finnmark	489	174	35.58	31.47	39.92
Agder	2537	872	34.37	32.55	36.24
Østfold	2167	686	31.66	29.73	33.65
Telemark	1125	349	31.02	28.39	33.79
Buskerud	1791	502	28.03	26.00	30.15

Table S6. Maternal RSV coverage stratified by district of residence in Oslo at time of delivery for women who delivered between 1 January and 30 September 2026, Norway

Oslo districts	Population	RSV Vaccinated			
	N	N	%	95%CI	
Nordre Aker	490	349	71.22	67.06	75.06
Vestre Aker	495	334	67.47	63.23	71.45
Sagene	621	390	62.8	58.93	66.51
Grünerløkka	808	458	56.68	53.24	60.06
Frogner	512	290	56.64	52.31	60.87
Ullern	365	203	55.62	50.49	60.63
St. Hanshaugen	388	206	53.09	48.12	58.00
Nordstrand	451	235	52.11	47.50	56.68
Gamle Oslo	793	387	48.8	45.34	52.28
Østernsjø	478	233	48.74	44.29	53.22
Bjerke	424	171	40.33	35.77	45.07
Grorud	193	47	24.35	18.84	30.87
Alna	378	87	23.02	19.06	27.52
Søndre Nordstrand	292	47	16.1	12.33	20.75
Stovner	224	32	14.29	10.30	19.47

Figure S1. Overview of socio-economic distribution among Oslo districts based on data from Statistics Norway. Data extracted on 15 October 2026 from https://www.ssb.no/statbank

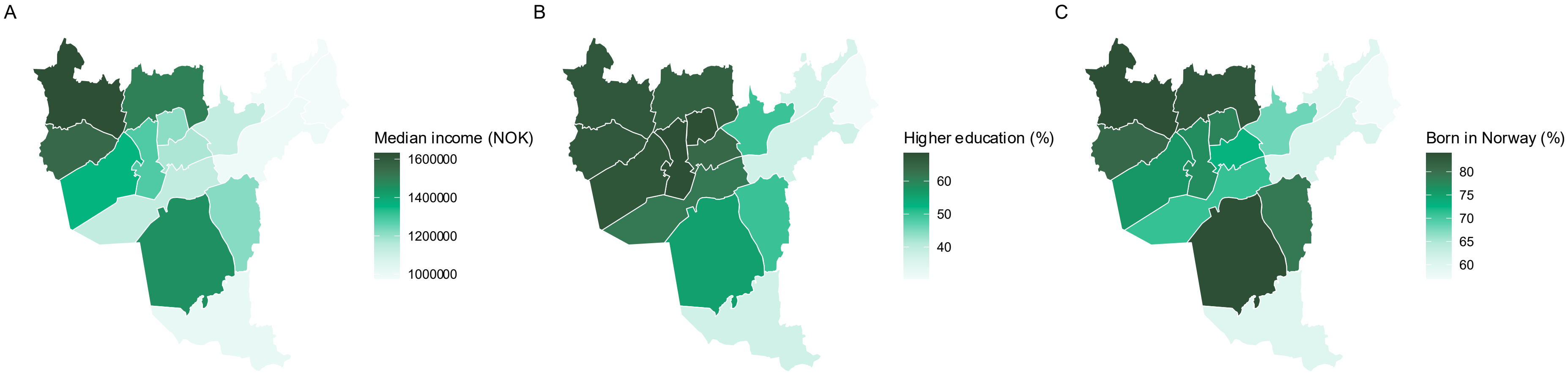

District	Income*	Cov (%)
Vestre Aker	1631700	71
Ullern	1554800	61
Nordre Aker	1493300	75
Nordstrand	1453100	57
Frogner	1359600	61
St. Hanshaugen	1284600	53
Østernsjø	1219400	53
Sagene	1212500	67
Grünerløkka	1173500	60
Gamle Oslo	1133600	52
Bjerke	1128700	45
Søndre Nordstrand	1013800	21
Alna	991500	28
Stovner	977000	19
Grorud	976500	31

**median income among couples with children under the age of 18*

District	Higher education (%)	Cov (%)
St. Hanshaugen	68.5	53
Sagene	68.4	67
Frogner	67.3	61
Vestre Aker	67.1	71
Ullern	66.8	61
Nordre Aker	65.5	75
Grünerløkka	64.1	60
Gamle Oslo	61.4	52
Nordstrand	55.6	57
Østernsjø	49.9	53
Bjerke	49.8	45
Alna	37.4	28
Søndre Nordstrand	37.3	21
Grorud	36.3	31
Stovner	30.4	19

District	Norwegian born (%)	Cov (%)
Vestre Aker	84	71
Nordstrand	84	57
Nordre Aker	83	75
Ullern	81	61
Østensjø	79	53
Sagene	78	67
St. Hanshaugen	77	53
Frogner	76	61
Grünerløkka	73	60
Gamle Oslo	71	52
Bjerke	68	45
Alna	61	28
Grorud	60	31
Søndre Nordstrand	60	21
Stovner	57	19
